# Successful treatment of nummular headache with Neurotropin^®^

**DOI:** 10.1007/s10194-011-0382-9

**Published:** 2011-09-09

**Authors:** Yuu Yamazaki, Keitaro Kobatake

**Affiliations:** 1 Department of Clinical Neuroscience and Therapeutics, Hiroshima University Graduate School of Biomedical Sciences, 1-2-3 Kasumi, Minami-ku, Hiroshima, 734-8551 Japan; 2 Department of Neurology, Kobatake Hospital, Fukuyama, Japan

Dear Sirs,

Nummular headache (NH) (or coin-shaped cephalalgia) is a chronic, mild-to-moderate, pressure-like pain in a small circumscribed area of the head in the absence of any lesion of the underlying structures, first described in 2002 [1]. While >200 cases have been reported so far, the treatment of NH is still a matter of debate. In this report, we describe a patient with NH who was successfully treated with Neurotropin^®^ (NTP), an analgesic drug currently used in Japan and China for the treatment of chronic pain conditions.

A 28-year-old man reported, for ~2-month period, continuous pressure-like pain of mild intensity in a rounded area 3–4 cm in diameter of the left parietal region. The pain tended to worsen in intensity during the evening, and it was accompanied by discomfort and increased sensitivity to light touch within the same area. No nausea, vomiting, photophobia, or phonophobia was reported. His past and family history was unremarkable. He had been taking over-the-counter analgesics and loxoprofen sodium prescribed from another hospital with no relief. Neurologic examination and cerebral computed tomography were normal.

In accordance with International Classification of Headache Disorders (ICHD)-II criteria, a diagnosis of NH was made. The patient was treated with NTP 16 units/day, which provided a significant pain relief within the next 2 weeks. He was followed 12 weeks later and reported that he stopped taking NTP after 2 months, without recurrence of NH.

NTP, a nonprotein extract from inflamed skin of rabbits inoculated with vaccinia virus, is widely used in Japan to treat chronic pain conditions such as lumbago, neck–shoulder–arm syndrome, fibromyalgia, and postherpetic neuralgia. In animal models, NTP showed anti-nociceptive effects via activation of the descending pain inhibitory pathway [2, 3]. Although it is unclear whether NH represents a focal, nociceptive-type pain stemming from epicranial tissues or neuralgia of a terminal branch of a pericranial sensitive nerve [4], the anti-nociceptive action of NTP seemed beneficial against NH in this patient.

We feel that the present observation adds NTP as a possible suitable choice of treatment for NH. Further to corroborate the efficacy of NTP, we hope that the results of a double-blind study of this agent in complex regional pain syndrome conducted at the US National Institutes of Health [5], which are expected shortly, would lead to approval of NTP in many other countries.

Informed consent for possible publication in The Journal of Headache and Pain was obtained from the patient.
